# Chromatic intervention and biocompatibility assay for biosurfactant derived from *Balanites aegyptiaca* (L.) Del

**DOI:** 10.1038/s41598-021-83573-7

**Published:** 2021-02-18

**Authors:** Vishal Panchariya, Vishal Bhati, Harishkumar Madhyastha, Radha Madhyastha, Jagdish Prasad, Priyal Sharma, Paras Sharma, Harish, Mahesh Kumar Saini, Vishnu D. Rajput, Yuichi Nakajima, S. L. Kothari, Vinod Singh Gour

**Affiliations:** 1 Amity Institute of Biotechnology, Amity University Rajasthan, Jaipur, India; 2 Department of Applied Physiology, School of Medicine, University of Miyazaki, Kiyotake-Cho, Kihara, Miyazaki, 5200 Japan; 3 Amity School of Applied Science, Amity University Rajasthan, Jaipur, India; 4 Food Chemistry Division, ICMR-National Institute of Nutrition, P/O Jamai Osmania, Tarnaka, Hyderabad, 500007 India; 5 Plant Biotechnology Laboratory, Department of Botany, Mohanlal Sukhadia University, Udaipur, 313 001 Rajasthan India; 6 Southern Federal University, Rostov-on-Don, 344090 Russia

**Keywords:** Biological techniques, Plant sciences, Materials science

## Abstract

Extraction of biosurfactants from plants is advantageous than from microbes. The properties and robustness of biosurfactant derived from the mesocarp of *Balanites aegyptiaca* have been reported. However, the dark brown property of biosurfactant and lack of knowledge of its biocompatibility limits its scope. In the present work, the decolorization protocol for this biosurfactant was optimized using hydrogen peroxide. The hemolytic potential and biocompatibility based on cell toxicity and proliferation were also investigated. This study is the first report on the decolorization and toxicity assay of this biosurfactant. For decolorization of biosurfactant, 3^4^ full factorial design was used, and the data were subjected to ANOVA. Results indicate that 1.5% of hydrogen peroxide can decolorize the biosurfactant most efficiently at 40 °C in 70 min at pH 7. Mitochondrial reductase (MTT) and reactive oxygen species (ROS) assays on M5S mouse skin fibroblast cells revealed that decolorized biosurfactant up to 50 µg/mL for 6 h had no significant toxic effect. Hemolysis assay showed ~ 2.5% hemolysis of human RBCs, indicating the nontoxic effect of this biosurfactant. The present work established a decolorization protocol making the biosurfactant chromatically acceptable. Biocompatibility assays confirm its safer use as observed by experiments on M5S skin fibroblast cells under in vitro conditions.

## Introduction

Surfactants (surface-active materials) belong to a diverse group of chemicals and may be divided into three categories, namely cationic, anionic and neutral, depending upon their polar moiety^1^. Currently, surfactants are manufactured from petrochemicals, and they are used in various industries like pharmaceuticals, detergents, cosmetics, petroleum, textile, food, agriculture, paper, and water treatment^2–4^. However, some harmful effects of synthetic surfactants present in shampoos have been observed, like hair loss, irritation to the scalp, and drying out of follicles^5,6^. Further, current strict environmental regulations across the countries and apprehensions among consumers for its safe use have drawn researchers' attention to exploring alternative options to synthetic surfactants^7^. The natural surfactants can be obtained easily from many marine organisms and various plants^8–11^. Biosurfactants possess high-foaming capacity, high-biodegradability, environment-friendly, low-toxicity, low-cost^12^, and par with chemically-synthesized surfactants^8^. They are also used for environmental clean-up like bioremediation of heavy metals and oil-recovery from water and soil^9,13^.

Commercially, *Pseudomonas* and *Bacillus* bacterial groups are the largest producers of biosurfactants^14,15^. *Lactococcus lactis* mediated cost-effective biosurfactant has also been reported^16^. Production of biosurfactants from bacteria is not cost-efficient due to cumbersome downstream processing, the requirement of a skilled workforce, and instrumentation for maintenance of bacterial strain under specific conditions. Besides, uncertainty in production output per batch is a point of concern^1^. Biosurfactants obtained from plants are proteins or protein hydrolysates, phospholipids, and saponins. Saponins have some biological and physiochemical properties and therefore are suitable for various biotechnological applications^1,5,11,17^. Saponins are nonionic and could be used in folk remedies and employed as detergents for textile cleaning^1,18^.

*Balanites aegyptiaca* has excellent detergent potential in its mesocarp. Its biosurfactant properties, including density, surface tension, viscosity, pH, electric conductivity, foaming potential, foam stability, detergency, dirt dispersal potential, and sebum removal potential, have been explored^19^. Sharma et al.^20^ reported the robustness of the detergent at varying pH, temperature, and salinity. However, the biosurfactant's brown color and a lack of biosafety evaluation limit its application in various industrial sectors. Decolorization and biocompatibility investigation of the biosurfactant can improve its market acceptance. Hydrogen peroxide (H_2_O_2_) is a chlorine-free oxidizing agent, and by providing free •OH radicals, it mediates the breakdown of several organic substances and bleaches the substances^21–24^. Bleaching efficiency depends on the dose of H_2_O_2_, the temperature, pH of the medium, and duration of the interaction^23,25^. Zhang and Zheng (2009) conducted experiments to optimize the decolorization of acid green dye (AG20) using various pH, H_2_O_2_ concentration, and ultrasonic power density. They found that the optimum conditions for decolorization of AG20 were 4.85 of initial pH, 1.94 mM of H_2_O_2,_ and 1.08 W/mL of ultrasonic power density^23^. Similarly, the decolorization process has also been optimized for polysaccharides obtained from *Cyclocarya paliurus*, where 0.5 mg/mL polysaccharides, 0.623 mM H_2_O_2_, 40℃ temperature, and 9.0 pH were found to be the best combination^24^.

Biosurfactants find applications in various fields such as cosmetics, skincare, laundry, utensils and floor cleaning, etc. Biosurfactants can enhance drug availability to the target cells and can facilitate the absorption of active principles. To test the biosurfactant for its efficacy in cosmetics, skincare, and pharmaceuticals, the in vitro toxicity assays are essential before conducting any in *vivo* tests. The toxicity assay includes preliminary tests like hemolysis and cell viability assays.

In the present study, the decolorization of biosurfactant derived from *B. aegyptiaca* was optimized using H_2_O_2_. Further, the toxicity of the biosurfactant was investigated by hemolysis and in vitro biocompatibility assays. The present investigation findings could be advantageous in developing a market acceptable, safe biosurfactant with a wide range of applications.

## Materials and methods

### Plant material

Mature fruits of *B. aegyptiaca* were collected from Jaipur and nearby areas (N 26°44.260′ E 075°43.174′ and 338 m altitude). After removing the pericarp, the mesocarp was collected by scratching the fruit surface with a sharp knife. This mesocarp was dried in a hot air oven at 65 °C for 48 h. The dried mesocarp was further powdered using the mortar-pestle and stored in an airtight container at room temperature until further use.

### Preparation of biosurfactant

Dried mesocarp powder (10 g) was mixed in 100 mL distilled water to get 10% (w/v) suspension of the biosurfactant.^19^ The suspension was shaken for 10 min and then filtered through blotting paper followed by Whatman No. 1 filter paper. The filtrate was used as the biosurfactant solution in further experiments.

### Decolorization of biosurfactant, experimental design, and statistical analysis

The biosurfactant solution (400 µL) was mixed with 20 µL H_2_O_2_ and 580 µL distilled water. Then the pH of this reaction mixture was adjusted to 6 using either 1 N NaOH or 1 N HCl. The final volume of this reaction mixture was raised to 4000 µL using distilled water. The reaction mixture was then incubated at 30℃ for 60 min (This is referred to as decolorization at 0.5% H_2_O_2,_ pH 6, 30℃ for 60 min incubation period). The mixture was cooled to room temperature, and the absorption of light at 495 nm wavelength (A_495_) was recorded using a spectrophotometer (Thermo Fisher Scientific, USA), taking distilled water as a blank. Reduction in absorbance was considered as a measure of decolorization. Experiments were carried out to evaluate the effect of four factors, namely dose of H_2_O_2_, pH, temperature, and duration of treatment on the decolorization (Supplementary Table S1). Each aspect was studied at three different levels (H_2_O_2_ at 0.5, 1.0 and 1.5% v/v; pH at 6, 7 and 8; the temperature at 30, 40, and 50 °C and duration of incubation of 60, 70, and 80 min, respectively; Supplementary Table S1) as described by Wang et al.^25^. A complete 3^4^ factorial experiment was conducted in a randomized block design with three replicates (blocks), resulting in 81 treatment combinations and 243 observations (Supplementary Table S1). The data were subjected to analysis of variance (ANOVA) for testing various hypotheses regarding the biosurfactant's decolorization. The main effects and interactions of factors were tested to determine the most optimum treatment combinations for decolorization of biosurfactant.

The biosurfactant was decolorized using 1.5% H_2_O_2_ at 40 °C for 70 min and tested for biocompatibility.

### Biosafety assay

#### Materials

M5S mouse skin fibroblast (JCRB1322) was obtained from the National Institute of Biomedical Innovation Health and Nutrition (NIBIOHN, Ibaraki, and Osaka), Japan. Cell culture medium alpha-MEM was purchased from Sigma Aldrich Co. (St. Louis, MO), USA. Fetal bovine serum (FBS) and PSN antibiotic cocktail mixture (penicillin 5 mg/mL, streptomycin 5 mg/mL, neomycin 10 mg/mL) were bought from Gibco Co Ltd (Gibco, Tokyo), Japan. Oxiselect TM intracellular ROS assay kit was procured from Cell Biolabs Inc. (San Diego, CA), USA. Cell viability and proliferation assay kit were purchased from Dojindo Laboratories (Dojindo, Molecular Technologies Inc. Kumamoto), Japan. All other analytical grade chemicals were bought from Wako (Wako analytical, Osaka) or Nacalai Tesque Inc. (Nacalai Tesque, Tokyo), Japan.

#### Methods

##### Cell toxicity assay

m5S skin fibroblast cells were cultured in alpha-MEM medium with 10% FBS and 1% PSN at 37 °C in a 5% CO_2_ animal cell incubator up to the sub-confluent stage. Cells were treated with different doses of biosurfactant (0, 5, 10, 25, 50, 100, and 200 µg/mL) for 12 h in 96 well culture plate under conditions as stated above. After the incubation period, cells were washed in cold (4℃) phosphate buffer saline (PBS), and MTT (1 mg/mL) was added to the wells and incubated further at 37 °C with standard incubation chamber for 3 h.

After the second incubation time, MTT was removed from each well, and 50 µL DMSO was added to dissolve the formazan crystal. The resulting intracellular formazan content was quantified by taking absorbance at 570 nm using a spectrophotometer (Multi-scan FC, Thermo Fisher Scientific, Inc., Pittsburg, PA, USA).

Percentage cell viability data were tested for normality based on the histogram, and data were not found normal. Then data were transformed to log10, and then they were subtracted from the highest value. These values were then subjected to a normality test, and the coefficients of skewness and kurtosis were found to be 0.376 and 0.921, respectively. Similarly, Kolmogorov–Smirnov and Shapiro–Wilk coefficient was found to be 0.082 and 0.024 (degree of freedom = 55). These findings proved that the data is not significantly different from normal data. Then the transformed data were subjected to one-way analysis of variance (ANOVA) at *p* < 0.05. F (48, 6) = 35.365, *p* = 0.000). Further, Duncan's multiple range test was applied to compare two treatment means (Degree of freedom = 6).

The decolorized biosurfactant effect on cell viability was also investigated. The percentage of cell viability was tested for normality based on the histograms, and data were found to be statistically normal (skewness = 0.391 and kurtosis = 1.639). Then the transformed data were subjected to one-way analysis of variance (ANOVA) at *p* < 0.05.

##### Cell proliferation assay

Sub confluent m5S cells were treated with biosurfactant (50 µg/mL) for various time intervals of 0, 2, 6, 10, 15, 24, and 48 h. Untreated cells at each time were used as the control group. Following incubation, the cells were processed by MTT assay to calculate the percentage of live cells, as per the manufacturer's instructions.

Data were subjected to a normality test and were found to be not significantly different from a normal distribution statistically. Then proliferation was compared after 6 and 10 h based on an independent sample t-test using SPSS version 16 at the significance level *P* < 0.05. Further analysis of skewness and kurtosis (6 h skewness z value: 0.289, 6 h kurtosis z value: 0.590, 10 h skewness z value: 0.2996, and 10 h kurtosis z value: 1.787) indicated that these values are in a range of ± 1.96, justifying that data is normal.

Similarly, the effect of decolorized biosurfactant (50 µg/mL) was also studied. The data obtained were subjected to the normality test and were found to be statistically normal. Then proliferation was compared after 6 and 10 h based on an independent sample t-test using SPSS ver. 16 at the significance level *P* < 0.05.

### Quantification of intracellular reactive oxygen species (ROS)

Intracellular reactive oxygen species (ROS) content was measured using a standard commercial kit (Cell Biolabs, Inc. San Diego, CA, USA). Cell population at 1 × 10^4^ cells/mL density were treated with different doses of biosurfactant (0, 5, 10, 25, 50, 100 and 200 µg/mL) for 6 h in 96 well culture plate. Following treatment, cells were washed with Hank's balanced salt solution (Gibco. Co. Ltd., Milliwake, USA) and incubated in 10 µM of dichlorofluorescin diacetate (Sigma. Aldrich. Inc. MO.USA) for 30 min at 37 °C. The fluorescence signal was quantified using a spectrophotometer (DTX800, Beckman Coulter, Inc. Brea, CA, and USA) at excitation and emission of 485 and 530 nm.

The same procedure was used to evaluate the influence of a 6 h incubation with the decolorized biosurfactant (0, 5, 10, 25, 50, 100, and 200 µg/mL) on cell viability.

The data obtained as percentage fluorescent units based on biosurfactant effect were subjected to normality test. They were not statistically significantly different from a normal distribution (Degree of freedom = 147, Kolmogorov–Smirnov coefficient = 0.086, skewness z value = 0.659, and kurtosis z value: 0.753). The data were further subjected to one-way analysis of variance (ANOVA) at *p* < 0.05 using SPSS ver. 16.

Similarly, the data on the effect of decolorized biosurfactants were also subjected to normality test. They were statistically significantly different from a normal distribution (Degree of freedom = 21, Sig. = 0.000, skewness z value = 1.258, and kurtosis z value: 0.200). The difference could be due to the small data size. The data were subjected to a non-parametric Kruskal–Wallis analysis of variance (ANOVA) test using SPSS ver. 16. The results (Chi-square test with Degree of freedom = 6, *p* = 0.003 < 0.005) indicated that the treatment with decolorized biosurfactants yielded statistically significant differences in cell viability percentage. The Mann–Whitney U test was applied using SPSS ver. 16 for comparison of each possible pair of treatments (21 pairs of treatments).

### Immuno-cytochemistry of DCF-DA (2′-7′dichlorofluorescin diacetate)

Cells (3 × 10^4^/mL) were seeded on culture compatible coverslips in 6 well culture plates and incubated for 6 h and 10 h with biosurfactant (0, 50 µg/mL) or decolorized biosurfactant (0, 50 µg/mL). The cells were washed with cold (4℃) PBS solution and fixed with 4% paraformaldehyde solution at room temperature for 10 min. After fixing, cells were again washed with PBS and mounted on glass slides. Intracellular green fluorescence was observed under a fluorescence microscope at excitation and emission of 485 and 530 nm, respectively, for 1/5 s (BZ-9000, Keyence, Osaka, Japan). The fluorescence intensity was obtained as histograms using the Image J software (Fig. 5).

### Haemolysis assay

Biosurfactant's in vitro toxicity was evaluated by a hemolytic assay using human red blood cells (RBCs) as described earlier^26^. This assay has been used to study the toxic nature of silver nanoparticles (AgNPs)^27^. The magnitude of hemolysis of RBCs indicates the level of toxicity of the material under study. Five percent biosurfactant solution was prepared by suspending the dried mesocarp powder in PBS and sterilized distilled water separately. The suspension was filtered through a syringe filter (0.22 µm). This filtrate was used as a biosurfactant solution. Approx. 10^8^ cells of RBCs were mixed with biosurfactant solution and incubated for 1 h at 37 °C under shaking conditions. The mixture was centrifuged at 2000 rpm for 10 min at room temperature to remove the debris. The absorbance of the supernatant was recorded at 540 nm. Triton X-100 (1% w/v) was used as a positive control. The percentage of hemolysis was calculated as follows:

$$\mathrm{\%\,of\,hemolysis}=\frac{\left(A-B\right)*100}{(C-B)}$$

where A, B, C are absorbance at 540 nm in the presence of biosurfactant, PBS, and 1% Triton X-100, respectively.

## Results and discussion

### Effect of variable parameters on decolorization of biosurfactant solution

The results obtained from various experiments to understand the effect of percentage H_2_O_2_, temperature, pH, and duration of treatment individually and in combination, are explained as follows.

#### Temperature

Temperature shows a highly significant effect on the decolorization of biosurfactant solution (Tables 1 and 2). Interaction of temperature parameter with other independent factors also indicates a significant impact. Homogenous subsets obtained by performing post-hoc test revealed that 40 and 50 °C contribute equally, but 50 °C contributes maximum (*p* ≤ 0.05) for the decolorization of biosurfactant (Table 1). Similar observations were recorded in decolorization of polysaccharides derived from *Cyclocarya paliurus,* where decolorization increased with an increase in temperature from 20 to 60 °C, with more than 80% decolorization efficiency at 40°C^24^. Salem et al.^28^ reported that an increase in temperature increased decolorization up to 40 °C. However, 30 °C is reported as an optimum condition for decolorization of acid blue dye.

**Table 1:** Descriptive statistics for the effect of temperature, time, pH and percentage hydrogen peroxide on decolorization of biosurfactant solution.

Treatment		Mean A_495_ ± SE
Temperature	30	0.3518 ± 0.18065
	40	0.2255 ± 0.06764
	50	0.2083 ± 0.05481
Duration	60	0.2549 ± 0.11100
	70	0.2663 ± 0.15593
	80	0.2644 ± 0.12624
Percentage hydrogen peroxide	0.5%	0.3016 ± 0.15255
	1%	0.2596 ± 0.08539
	1.5%	0.2244 ± 0.05939
pH	6	0.2895 ± 0.14385
	7	0.2673 ± 0.14875
	8	0.2287 ± 0.08565

**Table 2:** Effect of temperature, time duration, pH and hydrogen peroxide % on the decolorization of biosurfactant (ANOVA table for univariate analysis, *p* < 0.05).

Tests of between-subjects effects					
Dependent variable: absorbance at 495 nm					
Source	Type III Sum of squares	df	Mean square	F	Sig
Corrected model	3.169^a^	80	0.040	6.150	0.000
Intercept	16.662	1	16.662	2.587E3	0.000
Temperature	0.995	2	0.497	77.233	**0.000**
Time	0.006	2	0.003	0.473	0.624
Percentage H_2_O_2_	0.242	2	0.121	18.811	**0.000**
pH	0.153	2	0.077	11.913	**0.000**
Temperature × time	0.213	4	0.053	8.255	**0.000**
Temperature × percentage H_2_O_2_	0.072	4	0.018	2.783	**0.029**
Temperature × pH	0.067	4	0.017	2.582	**0.039**
Time × percentage H_2_O_2_	0.104	4	0.026	4.031	**0.004**
Time × pH	0.090	4	0.022	3.488	**0.009**
Percentage H_2_O_2_ × pH	0.076	4	0.019	2.965	**0.021**
Temperature × time × percentage H_2_O_2_	0.319	8	0.040	6.197	**0.000**
Temperature × time × pH	0.288	8	0.036	5.583	**0.000**
Temperature × percentage H_2_O_2_ × pH	0.249	8	0.031	4.842	**0.000**
Time × percentage H_2_O_2_ × pH	0.094	8	0.012	1.829	0.075
Temperature × time × Percentage H_2_O_2_ × pH	0.200	16	0.013	1.944	0.020
Error	1.043	162	0.006		
Total	20.874	243			
Corrected total	4.212	242			

^a^R Squared = 0.752 (Adjusted R Squared = 0.630)

#### Time duration

Different time durations (60, 70, and 80 min) were used to study the effect of time as an independent variable on decolorization of biosurfactant solution. However, the data did not reveal a significant impact (*p* = 0.624) (Table 2). On the other hand, interactions of the duration of treatments with temperature, pH, and doses of H_2_O_2_ yielded significant effects (Table 2). All three variables of these time-durations contributed equally to the decolorization of biosurfactant. But 80 min duration was the most effective towards decolorization at *p* ≤ 0.05 level of variation (Table 1). Similar results were reported for decolorization of polysaccharides derived from *C. paliurus,* where decolorization increased with time up to 40 min. No significant bleaching effect was found beyond this time^24^.

#### Effect of pH

The statistical analysis indicates that pH plays a significant role in decolorizing biosurfactant solution (Table 2). Further, it has also been found that its combinations with the other three factors are also crucial (Table 2). pH 8 was the most suitable for decolorization with a mean value of absorbance as 0.2887 (Table 1). These findings conform with earlier published scientific reports where decolorization increased with an increase in pH from 5 to 9 and decreased at pH 10. The highest decolorization efficiency (~ 77%) was observed at pH 9 in polysaccharides derived from *C. paliurus*^24^.

#### Hydrogen peroxide (H_2_O_2_)

The dose of H_2_O_2_ as an individual variable and its interaction with the three factors plays a significant role (Table 2). Among all the doses, 1.5% had the maximum effect on decolorization of biosurfactant solution (Table 1). Decolorization of polysaccharides derived from *C. paliurus* increased with an increase in H_2_O_2_ dosage, with maximum decolorization obtained at 0.623 mM H_2_O_2_^24^. An increase in H_2_O_2_ dose from 0.02 M to 0.4 M enhanced the decolorization for Acid Blue 29 dye^28^. Decolorization increased from ~ 30% to ~ 60% when the dose of H_2_O_2_ was increased from 0.2 to 1.6 mM^29^. H_2_O_2_ has various advantages as a bleaching agent, like high stability to decolorize different substances, can quickly destroy the dye, high efficiency, and does not require any special equipment.^25^ Decolorization of textile effluents is typically carried out using H_2_O_2_. Wang et al.^25^ reported efficient decolorization of *Sapindus mukorossi* pericarp (dark brown) using 2.5% H_2_O_2_ at pH 6 and 80 °C after 80 min incubation period.

#### Effect of combination of various treatments towards decolorization of biosurfactant solution

**Figure 1:**
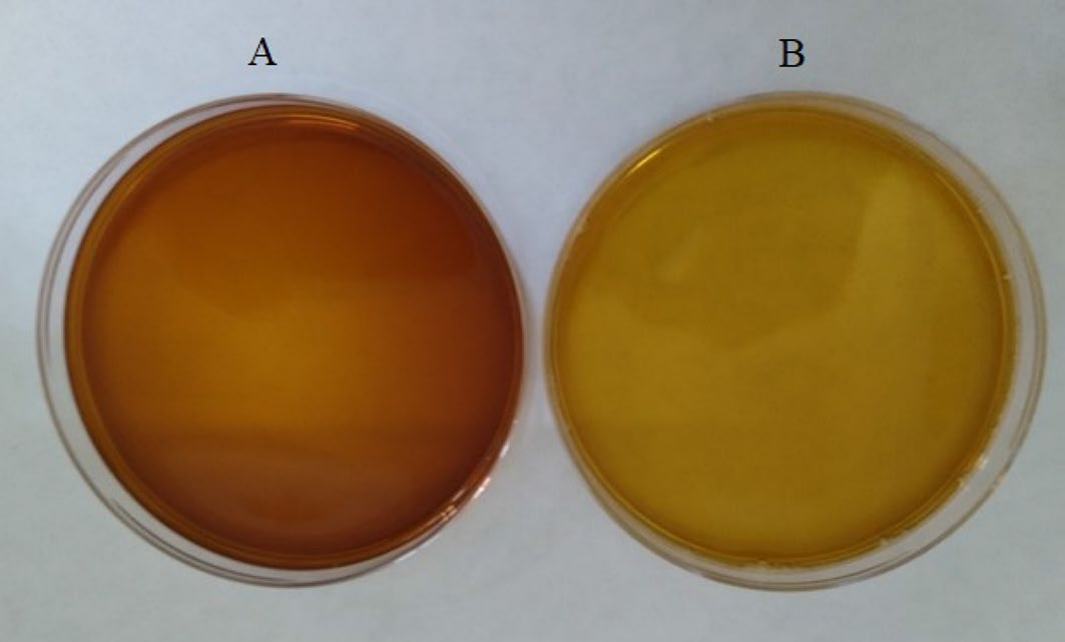
Biosurfactant solution: (**A**) before decolorization and (**B**) after decolorization.

The most effective combination towards decolorization was found with 50 °C and 60 min with a mean value of absorption of 0.180 (Table 3). Out of the nine combinations of temperature and percentage H_2_O_2_, the most effective combination towards decolorization was 40 °C and 1.5% H_2_O_2_ with a mean value of A_495_ of 0.183 (Table 3). Among the nine combinations of temperature and pH, 50 °C with pH 7 is the most effective combination towards decolorization with a mean value of A_495_ of 0.197 (Table 3). 70-min time duration with 1.5% H_2_O_2_ was the most effective combination with a minimum A_495_ of 0.218 (Table 3). Another combination of treatment duration and pH reveals that 60 min treatment at pH 8 was the most effective combination towards decolorization with a mean value of A_495_ of 0.211 (Table 3). Among the nine combinations of H_2_O_2_ and pH that were examined, 1.5% H_2_O_2_ at pH 8 was the most effective combination towards decolorization with a mean value of A_495_ of 0.207 (Table 3). In twenty-seven different combinations of temperature, duration of treatment, and percentage H_2_O_2_, the best combination was identified as 40 °C at 1.5% H_2_O_2_ for 80 min with the absorption of 0.159 (Table 3, Fig. 1). Among the twenty-seven different combinations of temperature, duration of treatment, and pH, the best combination was 50 °C for 60 min at pH 8 with the absorption of 0.160 (Table 3). Almost similar observation (0.161) has been observed at 40 °C for 80 min at pH 8 (Table 3). Among twenty-seven combinational approaches involving the duration of treatment, percentage H_2_O_2,_ and pH, the best combination was identified as 70 min treatment with 1.5% H_2_O_2_ at pH 7 with absorption of 0.187 (Table 3). Lastly, among the eighty-one combinations of temperature, duration of treatment, percentage H_2_O_2,_ and pH, the best combination has been identified as treatment with 1.5% H_2_O_2_ at pH 7 and 40 °C for 70 min with absorption of 0.139 (Table 3). From these observations, it can be inferred that the dose of H2O2 highly influences biosurfactant decolorization_._ 1.5% H_2_O_2_ treatment consistently resulted in better decolorization.

**Table 3:** Effect of combination of temperature, duration of treatment, percentage hydrogen peroxide and pH on decolorization of biosurfactant solution.

Temperature	Time duration (minutes)	Hydrogen peroxide (%)	pH	Mean A_495_	Range of A_495_
50	60	–	–	0.180	0.150–0.211
40	–	1.5	–	0.183	0.152–0.213
50	–	–	7	0.197	0.166–0.227
	70	1.5		0.218	0.187–0.248
–	60	–	8	0.211	0.181–0.242
–	–	1.5	8	0.207	0.176–0.237
40	80	1.5	–	0.159	0.107–0.212
50 (2)	60	–	8	0.160	0.108–0.213
40	–	1.5	8	0 .171	0.118–0.224
–	70	1.5	7	0.187	0.134–0.239
40	70	1.5	8	0.139	0.048–0.230

The data presented in this table are based on full factorial analysis.

### Biosafety aspects

#### In vitro cell toxicity assay

**Figure 2:**
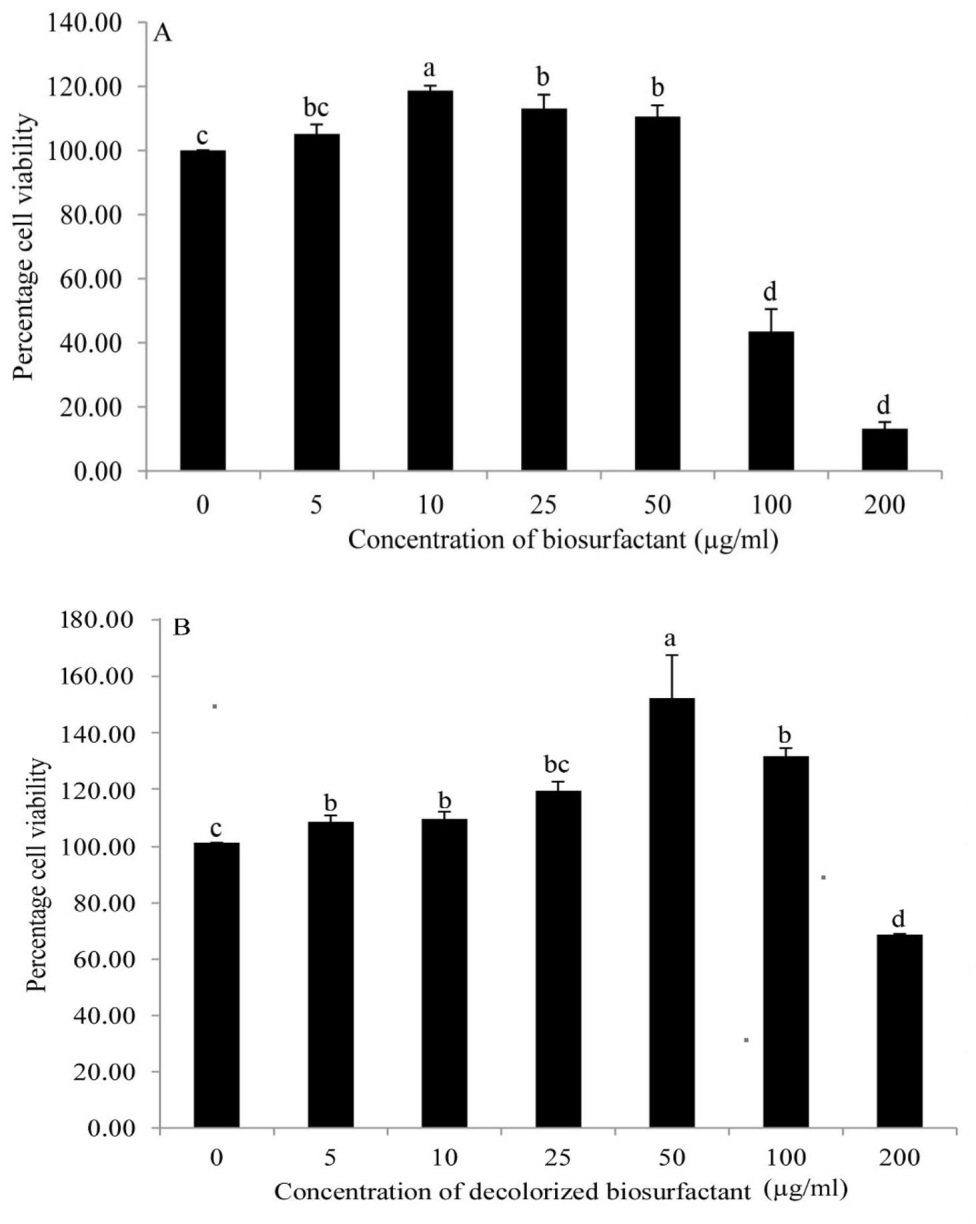
Toxicity assay of (**A**) biosurfactant and (**B**) decolorized biosurfactant at various concentrations on the mouse skin fibroblast (M5S) cell line (Results of the cell survival based on MTT assay). (Number of observations for biosurfactant and decolorized biosurfactant are 55 (df:6) and 21 (df:6) respectively. Error bars presents standard error. Similar letters given on the columns indicate that there is no statistically significant difference at *p* > 0.05) and the different letters on the column present statistically significant difference (*p* < 0.05) according to Duncan’s multiple range test.

**Figure 3:**
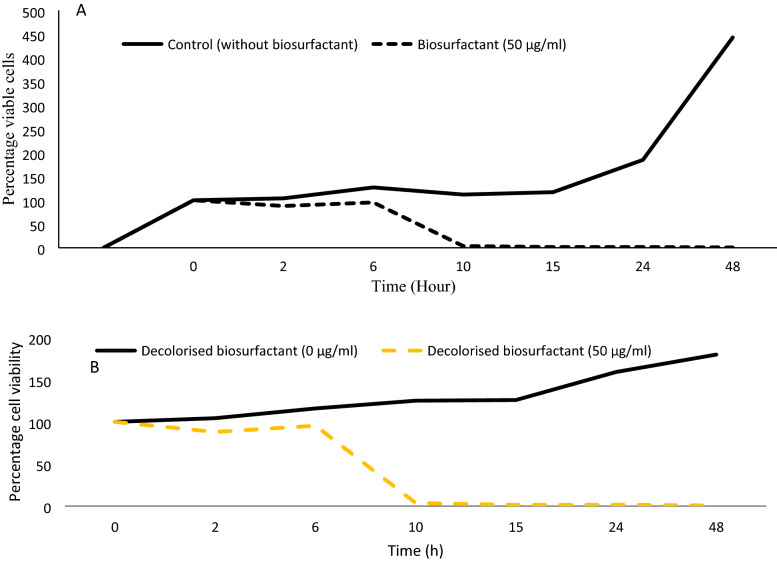
(**A**) Effect of biosurfactant on proliferation of mouse skin fibroblast (M5S) cell line (sample size:56) (**B**). Effect of decolorized biosurfactant on proliferation of mouse skin fibroblast (M5S) cell line. (**B**) (sample size: 42).

Observations recorded for MTT assay for cell viability have been presented in Fig. 2. It has been observed that there is no statistically significant difference in cell viability up to 50 µg/mL of biosurfactant treatment as observed in Fig. 2. Further, no significant difference in cell viability was found when cells were treated with 5 µg/mL, 25 µg/mL, and 50 µg/mL of biosurfactant with cell viability of 105%, 113%, and 110%, respectively. However, 100 µg/mL and 200 µg/mL of biosurfactant resulted in a drastic reduction in cell survival by 43% and 13%, respectively, indicating the biosurfactant's toxic effect on cells at higher concentration (Fig. 2). From these observations, it is inferred that the biosurfactant could be used up to 50 µg/mL without compromising in vitro cell viability. These results prove the safety of the material up to 50 µg/mL under in vitro conditions. The effect of 50 µg/mL biosurfactant and decolorized biosurfactant on cell proliferation was studied under different incubation times. The findings are presented in Fig. 3 A,B. Up to 6 h, the cell proliferation was similar between the groups, without statistically significant difference (*p* = 0.013, which is less than 0.05; Degree of freedom = 14). However, after 10 h, cell proliferation was significantly reduced (*p* = 0.000; Degree of freedom = 14). It can be concluded that the use of biosurfactant at 50 µg/mL is safe for in vitro cell proliferation. To our knowledge, there is no in vitro cell proliferation toxicity study available in the literature regarding aqueous extract of mesocarp (biosurfactant used in the present study) of *B. aegyptiaca*.

The results of cell viability following treatment with decolorized biosurfactant is presented in Fig. 2B. No significant difference in cell viability was observed on treatment with 0, 5, 10, 25, 100 µg/mL decolorized biosurfactant (Posthoc, Degree of freedom = 14). However, slightly better growth of cells was observed at 50 µg/mL treatment. Treatment with 200 µg/mL decolorized biosurfactant resulted in a drastic reduction in cell survival by ~ 66%, indicating its cytotoxic effect (Fig. 2B). Results show that up to 100 µg/mL decolorized biosurfactant could be used without compromising in vitro cell viability. It also proves the biocompatibility of decolorized biosurfactant up to 100 µg/mL under in vitro conditions. Decolorized biosurfactant (50 µg/mL) was used to study the effect of treatment duration on percentage cell viability (Fig. 3B). The findings indicate that up to 6 h of the incubation period, the proliferation of cells was similar to that of treatment with 50 µg/mL, without statistically significant difference (Levene's test is applied to test the equality of variances, F (2,2) = 0.029, *p* = 0.873 > 0.05). The T-test showed that the difference between control and 50 µg/mL was not statistically significant (t = 0.391, degree of freedom = 4, *p* = 0.716 > 0.05). It can be inferred that 50 µg/mL decolorized biosurfactant does not pose any cytotoxic effect. The ethanolic extracts of *B. aegyptiaca* fruit mesocarp did not exhibit any significant toxic effect when given orally at a concentration up to 4000 mg/kg body weight to Wistar albino rats^30^. The aqueous extract of mesocarp of *B. aegyptiaca* exhibited molluscicidal activities with LC_50_ values 65.51 mg/L and 83.52 mg/L, respectively, against *Biomphalaria pfeifferi* and *Lymnaea natalensis*^31^. A strong correlation between the saponin content of *B. aegyptiaca* mesocarp extracts and *Aedes aegypti* larval mortality was revealed^32^. Various studies have highlighted the presence of saponins, balanitoside and diosgenyl saponins, terpenoides, phenolic compounds and alkaloids^32–37^. These biochemicals may have direct and indirect effects on the survival of cells.

#### Reactive oxygen species (ROS)

**Figure 4:**
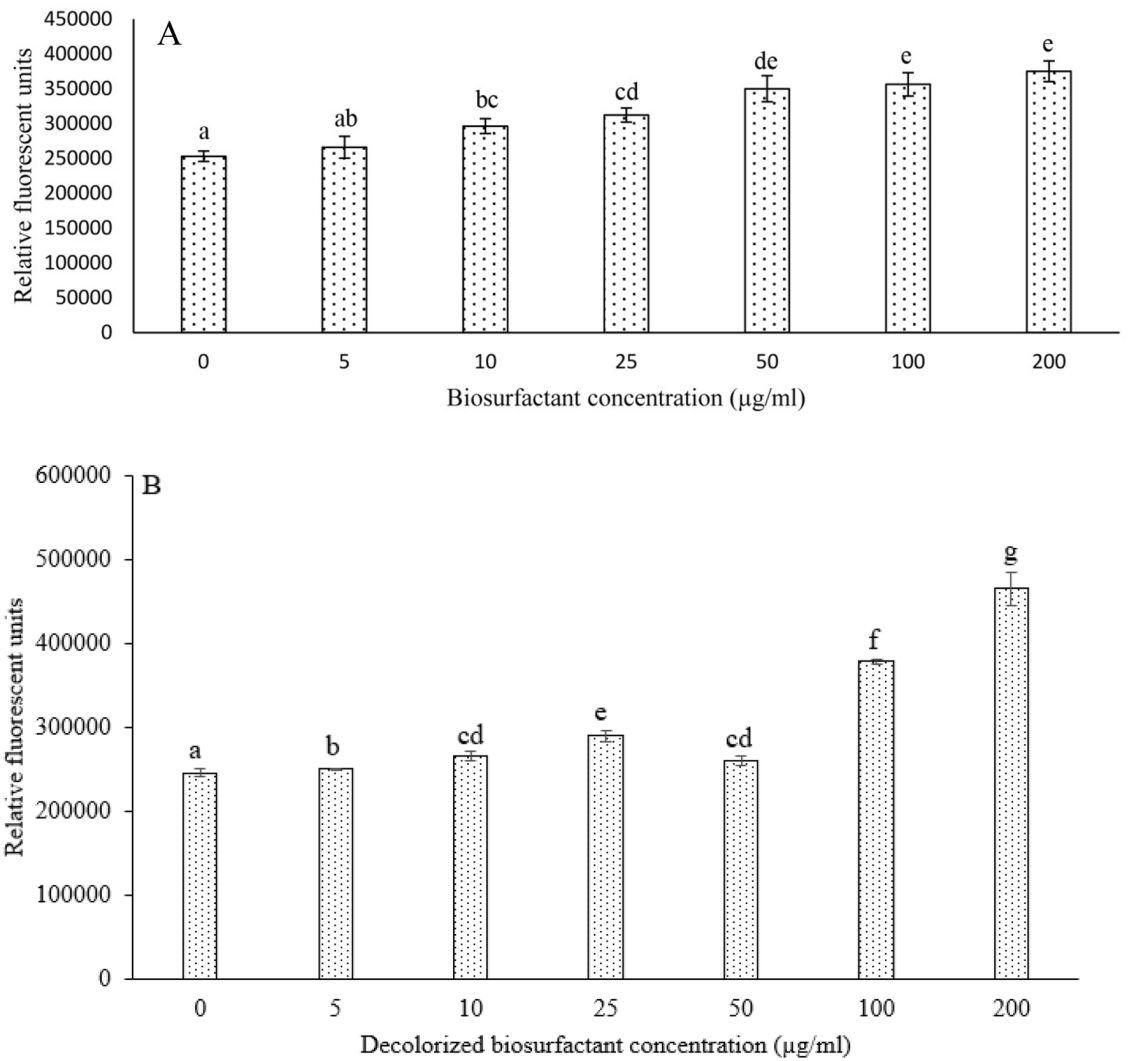
(**A**) Effect of biosurfactant on generation of reactive oxygen species (in terms of relative fluorescent units) on mouse skin fibroblast (M5S) cell line (sample size is147, Duncan’s multiple range test at df = 6) and (**B**) effect of decolorized biosurfactant on generation of reactive oxygen species (in terms of relative fluorescent units) on mouse skin fibroblast (M5S) cell line (sample size is 21, non-parametric Mann–Whitney test). Error bars in the graphs presents standard deviation. Similar letters on the columns indicate that there is no statistically significant difference at *p* > 0.05. Whereas dissimilar letters on the columns indicate that there is statistically significant difference at *p* < 0.05.

Biosurfactants induce ROS formation, which oxidizes the reduced form of DCFH_2_, resulting in DCFH_2_-mediated fluorescence. Fibroblast cells were treated with biosurfactant ( 0 µg/mL to 200 µg/mL) and evaluated for intracellular ROS generation (Fig. 4). The ROS was recorded in terms of relative fluorescent units. Biosurfactant at 5 µg/mL did not produce any significant ROS quantity (Fig. 4). An increase in the dose of biosurfactant increased the ROS generation (Fig. 4). Overall, the effect seems significant from a cytotoxicity perspective when 50 µg/mL biosurfactant was used. Again, the inference remains towards safe use of biosurfactant at a dose of 50 µg/mL. There is little literature available regarding the cytotoxicity of biosurfactants. Treatment of HepG2 cells with aqueous fruit extract of *B. aegyptiaca* resulted in intrinsic cell death mediated by down-regulation of the BCL2 gene^37^. Similarly, Beit-Yannai et al.^38^ reported that steroidal saponin extracted from roots and fruits of *B. aegyptiaca* caused the generation of ROS in h MCF-7 breast and HT-29 human colon cancer cells.

**Figure 5:**
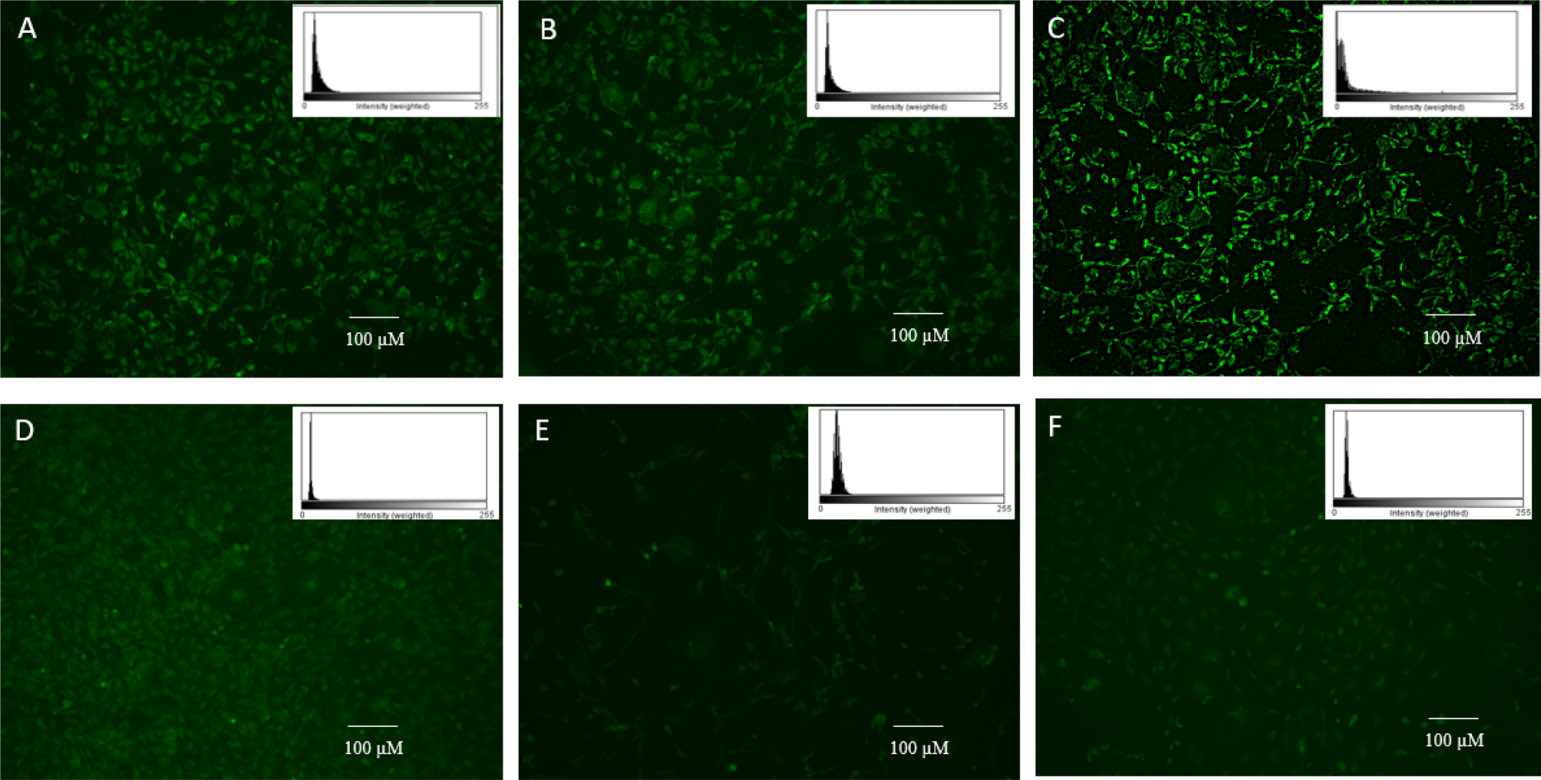
Intracellular ROS analysis by DCFH-Da Fluorescence assay. (**A**–**C**) Biosurfactant. (**A**) Wild control group. (**B**) cells treated with 50 µg/mL for 6 h and (**C**) 50 µg/mL for 10 h. (**D**–**F**) Decolorized biosurfactant. (**D**) Wild control group. (**E**) Cells treated with 50 µg/mL for 6 h and (F) 50 µg/mL for 10 h. Intensity based histogram (as calculated by image J) is presented in inset for each treatment.

Fibroblast cells were treated with different concentrations of decolorized biosurfactant and analyzed for ROS generation (Fig. 4B). The results indicate that decolorized biosurfactant had a statistically significant effect on ROS quantity based on Mann–Whitney U tests (coefficient: 0.000 < 0.05) except for one pair of treatments (10 µg/mL and 50 µg/mL, Mann–Whitney U tests coefficient: 1.000 > 0.05). As the concentration of decolorized biosurfactant increased, the ROS generation amount also increased, as evident from the correlation coefficient of 0.965. The decolorization process does not pose any significant toxic effect, as evident from the treatment up to 50 µg/mL (Fig. 5A,B).

The treatment of cells for 6 h with 50 µg/mL biosurfactant resulted in ROS generation (Fig. 5A,B). However, the amount of fluorescence increased after 10 h incubation significantly, as presented in Fig. 5C. It reflects that an increase in treatment duration enhances the increment in ROS generation and cell toxicity. Therefore, it can be inferred that cells should be safe if exposed to biosurfactants for ≤ 6 h.

The treatment of cells with 50 µg/mL decolorized biosurfactant resulted in ROS generation, with similar fluorescence intensity after 6 h and 10 h of incubation compared to the untreated cells (Fig. 5D–F). Therefore, it can be inferred that exposure to biosurfactant for ≤ 6 h and decolorized biosurfactant up to 10 h at 50 µg/mL concentration is not toxic to cells. The intensity of fluorescence obtained through Image J analysis indicates a significant increase in fluorescence intensity when cells are incubated with biosurfactant for 10 h at 50 µg/mL. However, there was no considerable accumulation of ROS in cells exposed to decolorized biosurfactant at 50 µg/mL up to 10 h, as evident from the histograms (inset of Fig. 5D–F).

#### Haemolysis assay

**Figure 6:**
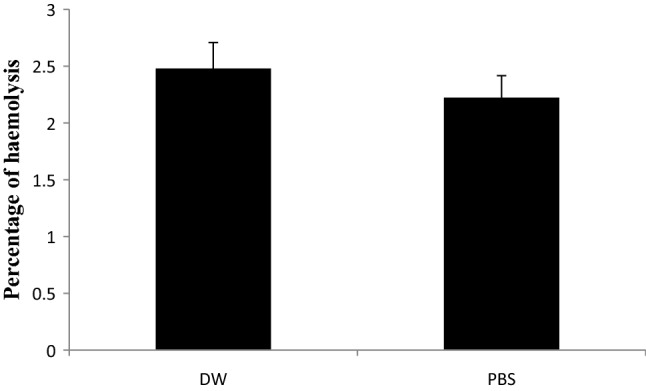
Percentage haemolysis by biosurfactant in distilled water and phosphate buffer saline (PBS). Number of observations: 6, Error bars in the graphs represents standard error.

The observations recorded as percentage hemolysis due to biosurfactant treatment are presented in Fig. 6. It indicates that biosurfactant in DW and PBS causes 2.48% and 2.22% hemolysis, respectively. While 100% hemolysis was observed in Triton X-100 (1%), no hemolysis was observed in DW. It indicates that the biosurfactant is relatively safer as the safer range is below 5% haemolysis.^39^.

## Conclusion

The advantages of biosurfactants over synthetic detergents have attracted researchers to the exploration of newer sources. The present investigation is the first study to reveal the cytosafety property of biosurfactant and decolorized biosurfactant derived from the mesocarp of *B. aegyptiaca*. Experiments were conducted to decolorize the biosurfactant to make it more acceptable to the market. The findings revealed that the most suitable combination of the factors studied is biosurfactant treatment with 1.5% H_2_O_2_ at 40 °C and pH 7 for 70 min. These findings bring out a strong base for in *vivo* study to explore this biosurfactant application as shampoo/detergent (hair growth, hair fall, microbial infection, etc.), laundry detergent, dishwasher, etc. Neutral pH, easy and economical method bleaching of the biosurfactant and retention of nontoxic nature (at 50 µg/mL for 6 h under in vitro conditions) even after decolorization of the present biosurfactant opens a wide range of applications of the biosurfactant derived from the mesocarp of *B. aegyptiaca.* It may find pharmacology applications as drug formulation components that can facilitate drug delivery and better access of the drug to the target cells. Further studies are required to establish applications of this biosurfactant in various fields.

## Supplementary Information

Supplementary Information.

## Abbreviations

ANOVA: Analysis of variance
DCF: 2′,7′-Dichlorofluorescin
DCF-DA: 2′-7′Dichlorofluorescin diacetate
DMSO: Dimethyl sulfoxide
DW: Distilled water
FBS: Fetal bovine serum
H_2_O_2_: Hydrogen peroxide
MTT: Mitochondrial reductase
PBS: Phosphate buffer saline
PSN: Penicillin, streptomycin, and neomycin
ROS: Reactive oxygen species

## References

[1] Bezerra KGO, Rufino RD, Luna JM, Sarubbo LA (2018). Saponins and microbial biosurfactants: Potential raw materials for the formulation of cosmetics. Biotechnol. Prog..

[2] Elazzazy AM, Abdelmoneim TS, Almaghrabi OA (2015). Isolation and characterization of biosurfactant production under extreme environmental conditions by alkali-halo-thermophilic bacteria from Saudi Arabia. Saudi. J. Biol. Sci..

[3] Varjani SJ, Upasani VN (2017). Critical review on biosurfactant analysis, purification and characterization using rhamnolipid as a model biosurfactant. Bioresour. Technol..

[4] Mahamallik P, Pal A (2017). Degradation of textile wastewater by modified photo-Fenton process: Application of Co (II) adsorbed surfactant-modified alumina as heterogeneous catalyst. J. Environ. Chem. Eng..

[5] Zhou W, Wang X, Chen C, Zhu L (2013). Enhanced soil washing of phenanthrene by a plant-derived natural biosurfactant, *Sapindus saponin*. Colloids. Surf. A.

[6] Sousa JR, Correia JA, Melo VM, Gonçalves LR, Cruz AJ (2014). Kinetic and characterization of rhamnolipid produced by *Pseudomonas aeruginosa* MSIC02 using glycerol as carbon source. Quim. Nova..

[7] Nitschke M, Pastore GM (2002). Biossurfactantes: propriedades e aplicações. Quim. Nova..

[8] Akbari S, Abdurahman NH, Yunus RM, Fayaz F, Alara OR (2018). Biosurfactants—a new frontier for social and environmental safety: A mini review. Biotechnol. Res. Innov..

[9] Boruah B, Gogoi M (2013). Plant based natural surfactants. Asian. J. Home. Sci..

[10] Cheeke PR, Oleszek W, Marston A (2000). Actual and potential applications of *Yucca schidigera* and *Quillaja saponaria* saponins in human and animal nutrition. Saponins in Food, Feedstuffs and Medicinal Plants.

[11] Cheok CY, Salman HAK, Sulaiman R (2014). Extraction and quantification of saponins: A review. Food. Res. Int..

[12] Smułek W, Zdarta A, Pacholak A, Zgoła-Grześkowiak A, Marczak L, Jarzębski M, Kaczorek E (2017). *Saponaria officinalis* L. extract: Surface active properties and impact on environmental bacterial strains. Colloids. Surf. B.

[13] Al-Wahaibi Y, Joshi S, Al-Bahry S, Elshafie A, Al-Bemani A, Shibulal B (2014). Biosurfactant production by *Bacillus subtilis* B30 and its application in enhancing oil recovery. Colloids. Surf. B.

[14] Sobrinho HB, Luna JM, Rufino RD, Porto ALF, Sarubbo LA, Govil JN (2013). Biosurfactants: classification, properties and environmental applications. Recent Developments in Biotechnology.

[15] Bezza FA, Chirwa EMN (2015). Production and applications of lipopeptide biosurfactant for bioremediation and oil recovery by *Bacillus subtilis* CN2. Chem. Eng. J..

[16] Vera ECS, de Azevedo PODS, Domínguez JM, de Souza Oliveira RP (2018). Optimization of biosurfactant and bacteriocin-like inhibitory substance (BLIS) production by *Lactococcus lactis* CECT-4434 from agroindustrial waste. Biochem. Eng. J..

[17] Sparg S, Light ME, Van Staden J (2004). Biological activities and distribution of plant saponins. J. Ethnopharmacol..

[18] Augustin JM, Kuzina V, Andersen SB, Bak S (2011). Molecular activities, biosynthesis and evolution of triterpenoid saponins. Phytochemistry.

[19] Gour VS, Sanadhya N, Sharma P, Parmar A, Datta M (2015). Biosurfactant characterization and its potential to remove sebum from hair. Ind. Crops. Prod..

[20] Sharma P, Saini MK, Prasad J, Gour VS (2019). Evaluation of robustness of the biosurfactant derived from *Balanites aegyptiaca* (L.) Del. J. Surfactants. Deterg..

[21] Dulman V, Ignat ME, Ignat L, Gânju D, Popa VI (2017). Decolorization of chlorolignin with hydrogen peroxide in the presence of silica [bis (dibenzoylmethido) copper ii] as catalyst. Environ. Eng. Manag. J..

[22] Fathima NN, Aravindhan R, Rao JR, Nair BU (2008). Dye house wastewater treatment through advanced oxidation process using Cu-exchanged Y zeolite: A heterogeneous catalytic approach. Chemosphere.

[23] Zhang Z, Zheng H (2009). Optimization for decolorization of azo dye acid green 20 by ultrasound and H_2_O_2_ using response surface methodology. J. Hazard. Mater..

[24] Xie JH, Shen MY, Nie SP, Li C, Xie MY (2011). Decolorization of polysaccharides solution from *Cyclocarya paliurus* (Batal.) Iljinskaja using ultrasound/H2O2 process. Carbohydr. Polym..

[25] Wang N, Wang H, Weng Z, Zhang C, Wu H, Guo Y, Yao W (2014). Decolorization of *Sapindus pericarp* extract by hydrogen peroxide and a comparison of basic characteristics before and after decolorization. J. Surfactants. Deterg..

[26] Liao KH, Lin YS, Macosko CW, Haynes CL (2011). Cytotoxicity of graphene oxide and graphene in human erythrocytes and skin fibroblasts. ACS Appl. Mater. Inter..

[27] Katva S, Das S, Moti HS, Jyoti A, Kaushik S (2017). Antibacterial synergy of silver nanoparticles with gentamicin and chloramphenicol against *Enterococcus faecalis*. Pharmacogn. Mag..

[28] Salem IA, El-Ghamry HA, El-Ghobashy MA (2014). Catalytic decolorization of acid blue 29 dye by H_2_O_2_ and a heterogeneous catalyst. Beni-Suef Univ. J. Basic Appl. Sci..

[29] Osuji AC, Eze SOO, Osayi EE, Chilaka FC (2014). Biobleaching of industrial important dyes with peroxidase partially purified from garlic. Sci. World J..

[30] Mhya DH, Amigo KM, Umar IA, Alegbejo JO (2016). Evalaution of hypoglycemic potential of extracts of *Balanites aegyptiaca* parts. Int. J. Innov. Adv. Stud..

[31] Molla E, Giday M, Erko B (2013). Laboratory assessment of the molluscicidal and cercariacidal activities of *Balanites aegyptiaca*. Asian Pac. J. Trop. Biomed..

[32] Wiesman Z, Chapagain BP (2006). Larvicidal activity of saponin containing extracts and fractions of fruit mesocarp of *Balanites aegyptiaca*. Fitoterapia.

[33] Abdallah EM, Hsouna AB, Al-Khalifa KS (2012). Antimicrobial, antioxidant and phytochemical investigation of *Balanites aegyptiaca* (L.) Del edible fruit from Sudan. Afr. J. Biotechnol..

[34] Stærk D, Chapagain BP, Lindin T, Wiesman Z, Jaroszewski JW (2006). Structural analysis of complex saponins of *Balanites aegyptiaca* by 800 MHz 1H NMR spectroscopy. Magn. Reson. Chem..

[35] Gnoula C, Mégalizzi V, De Nève N, Sauvage S, Ribaucour F, Guissou P, Kiss R (2008). Balanitin-6 and-7: Diosgenyl saponins isolated from *Balanites aegyptiaca* Del. display significant anti-tumor activity in vitro and in vivo. Int. J. Oncol..

[36] Pettit GR, Doubek DL, Herald DL, Numata A, Takahasi C, Fujiki R, Miyamoto T (1991). Isolation and structure of cytostatic steroidal saponins from the African medicinal plant *Balanites aegyptica*. J. Nat. Prod..

[37] Yassin AM, El-Deeb NM, Metwaly AM, El Fawal GF, Radwan MM, Hafez EE (2017). Induction of apoptosis in human cancer cells through extrinsic and intrinsic pathways by *Balanites aegyptiaca* furostanol saponins and saponin-coated silver nanoparticles. Appl. Biochem. Biotechnol..

[38] Beit-Yannai E, Ben-Shabat S, Goldschmidt N, Chapagain BP, Liu RH, Wiesman Z (2011). Antiproliferative activity of steroidal saponins from *Balanites aegyptiaca*—an *in vitro* study. Phytochem. Lett..

[39] Venkatesan B, Subramanian V, Tumala A, Vellaichamy E (2014). Rapid synthesis of biocompatible silver nanoparticles using aqueous extract of *Rosa damascena* petals and evaluation of their anticancer activity. Asian Pac. J. Trop. Med..

