# Supplementary material for: Chromatic intervention and biocompatibility assay for biosurfactant derived from Balanites aegyptiaca (L.) Del
Source: Sci Rep. 2021 Feb 18;11:4186. doi: 10.1038/s41598-021-83573-7 (PMC7893048; doi:10.1038/s41598-021-83573-7)
Supplement: Supplementary file 1 — Supplementary Information. [file 41598_2021_83573_MOESM1_ESM.docx]

**Supplementary table 1**.

Experimental design to study the effect of factors on decolorization of biosurfactant (each experiment was repeated thrice so 243 observations were recorded as Absorbance at 495nm)

| S No | pH | Percentage H_2_O_2_ | Temperature (℃) | Duration of treatment (Min.) |
| --- | --- | --- | --- | --- |
| 1 | 6 | 0.5 | 30 | 60 |
| 2 | 6 | 0.5 | 30 | 70 |
| 3 | 6 | 0.5 | 30 | 80 |
| 4 | 7 | 0.5 | 30 | 60 |
| 5 | 7 | 0.5 | 30 | 70 |
| 6 | 7 | 0.5 | 30 | 80 |
| 7 | 8 | 0.5 | 30 | 60 |
| 8 | 8 | 0.5 | 30 | 70 |
| 9 | 8 | 0.5 | 30 | 80 |
| 10 | 6 | 0.1 | 30 | 60 |
| 11 | 6 | 0.1 | 30 | 70 |
| 12 | 6 | 0.1 | 30 | 80 |
| 13 | 7 | 0.1 | 30 | 60 |
| 14 | 7 | 0.1 | 30 | 70 |
| 15 | 7 | 0.1 | 30 | 80 |
| 16 | 8 | 0.1 | 30 | 60 |
| 17 | 8 | 0.1 | 30 | 70 |
| 18 | 8 | 0.1 | 30 | 80 |
| 19 | 6 | 1.5 | 30 | 60 |
| 20 | 6 | 1.5 | 30 | 70 |
| 21 | 6 | 1.5 | 30 | 80 |
| 22 | 7 | 1.5 | 30 | 60 |
| 23 | 7 | 1.5 | 30 | 70 |
| 24 | 7 | 1.5 | 30 | 80 |
| 25 | 8 | 1.5 | 30 | 60 |
| 26 | 8 | 1.5 | 30 | 70 |
| 27 | 8 | 1.5 | 30 | 80 |
| 28 | 6 | 0.5 | 40 | 60 |
| 29 | 6 | 0.5 | 40 | 70 |
| 30 | 6 | 0.5 | 40 | 80 |
| 31 | 7 | 0.5 | 40 | 60 |
| 32 | 7 | 0.5 | 40 | 70 |
| 33 | 7 | 0.5 | 40 | 80 |
| 34 | 8 | 0.5 | 40 | 60 |
| 35 | 8 | 0.5 | 40 | 70 |
| 36 | 8 | 0.5 | 40 | 80 |
| 37 | 6 | 0.1 | 40 | 60 |
| 38 | 6 | 0.1 | 40 | 70 |
| 39 | 6 | 0.1 | 40 | 80 |
| 40 | 7 | 0.1 | 40 | 60 |
| 41 | 7 | 0.1 | 40 | 70 |
| 42 | 7 | 0.1 | 40 | 80 |
| 43 | 8 | 0.1 | 40 | 60 |
| 44 | 8 | 0.1 | 40 | 70 |
| 45 | 8 | 0.1 | 40 | 80 |
| 46 | 6 | 1.5 | 40 | 60 |
| 47 | 6 | 1.5 | 40 | 70 |
| 48 | 6 | 1.5 | 40 | 80 |
| 49 | 7 | 1.5 | 40 | 60 |
| 50 | 7 | 1.5 | 40 | 70 |
| 51 | 7 | 1.5 | 40 | 80 |
| 52 | 8 | 1.5 | 40 | 60 |
| 53 | 8 | 1.5 | 40 | 70 |
| 54 | 8 | 1.5 | 40 | 80 |
| 55 | 6 | 0.5 | 50 | 60 |
| 56 | 6 | 0.5 | 50 | 70 |
| 57 | 6 | 0.5 | 50 | 80 |
| 58 | 7 | 0.5 | 50 | 60 |
| 59 | 7 | 0.5 | 50 | 70 |
| 60 | 7 | 0.5 | 50 | 80 |
| 61 | 8 | 0.5 | 50 | 60 |
| 62 | 8 | 0.5 | 50 | 70 |
| 63 | 8 | 0.5 | 50 | 80 |
| 64 | 6 | 0.1 | 50 | 60 |
| 65 | 6 | 0.1 | 50 | 70 |
| 66 | 6 | 0.1 | 50 | 80 |
| 67 | 7 | 0.1 | 50 | 60 |
| 68 | 7 | 0.1 | 50 | 70 |
| 69 | 7 | 0.1 | 50 | 80 |
| 70 | 8 | 0.1 | 50 | 60 |
| 71 | 8 | 0.1 | 50 | 70 |
| 72 | 8 | 0.1 | 50 | 80 |
| 73 | 6 | 1.5 | 50 | 60 |
| 74 | 6 | 1.5 | 50 | 70 |
| 75 | 6 | 1.5 | 50 | 80 |
| 76 | 7 | 1.5 | 50 | 60 |
| 77 | 7 | 1.5 | 50 | 70 |
| 78 | 7 | 1.5 | 50 | 80 |
| 79 | 8 | 1.5 | 50 | 60 |
| 80 | 8 | 1.5 | 50 | 70 |
| 81 | 8 | 1.5 | 50 | 80 |
